# Aerodynamic measurements in singers at usual, low, and high frequencies

**DOI:** 10.1590/2317-1782/e20250325en

**Published:** 2026-05-11

**Authors:** Dâmaris Poliana Lacerda Vieira, Viviane Souza Bicalho Bacelete, Bárbara Pereira Lopes, Patrícia de Freitas Lopes Genilhú, Ana Cristina Côrtes Gama

**Affiliations:** 1 Programa de Pós-graduação em Ciências Fonoaudiológicas, Departamento de Fonoaudiologia, Faculdade de Medicina, Universidade Federal de Minas Gerais – UFMG - Belo Horizonte (MG), Brasil.

**Keywords:** Acoustics, Music, Singing, Respiration, Voice

## Abstract

**Purpose:**

To analyze aerodynamic measurements at habitual, low, and high frequencies in male and female singers without vocal changes.

**Methods:**

This is a cross-sectional, analytical, observational study with 30 male and 30 female singers, aged 19 to 48 years. Aerodynamic parameters such as air pressure, expired and voiced airflow, expiratory volume, power and aerodynamic resistance, acoustic impedance, and aerodynamic efficiency were measured by emitting the syllable /pá/ seven consecutive times at usual intensity and usual, low, and high frequencies.

**Results:**

Modifications in aerodynamic measurements during the emission of different vocal frequencies were more evident in male singers. They had higher values ​​of peak air pressure, aerodynamic resistance, acoustic impedance, and aerodynamic efficiency in high-pitched emissions, while female singers had higher values ​​of aerodynamic efficiency in usual emissions, followed by high-pitched emissions.

**Conclusion:**

The variation in fundamental frequency of the voice causes greater changes in aerodynamic parameters in male singers. Female singers had higher values ​​of aerodynamic efficiency in usual voice production.

## INTRODUCTION

Several theories about voice production have emerged over time, most notably Van Den Berg's aerodynamic-myoelastic theory, which interrelates the elasticity of the laryngeal muscles with the aerodynamic force of breathing, forming an interdependent system^(1)^. Ignoring any part of this system can compromise the balance in voice production and increase the risk of developing voice disorders, due to homeostasis breakdown^(1,2)^.

Voice clinics have increasingly used aerodynamic assessment, as these measures are part of the instrumental voice analysis recommendation protocol suggested by the American Speech-Language-Hearing Association (ASHA) and provide valuable functional information about the interrelationship between the phonatory and respiratory systems^(2,3)^. Lung volumes and capacities, subglottal air pressure (Psub), laryngeal airflow, laryngeal resistance, and s/z ratio are measures available to voice clinics, useful for providing details of phonation physiology^(2-4)^.

The interdependent mode of functioning of the phonatory system allows us to understand how the activity of the adductor muscles of the vocal folds (VF) can be directly affected by the aerodynamic process of phonation^(1,4,5)^. The Psub resulting from lung compression by means of the expiratory musculature and the closing force of the VF, which confers greater or lesser resistance to this transglottic airflow, serves as a source of energy for VF vibration and influences the intensity and variations of the fundamental frequency (f_0_) of the voice^(6-8)^.

Singing elicits several physical-acoustic parameters that are in many ways different from speech^(9,10)^. Physiologically, these mechanisms manifest in the control and interaction between Psub, glottal closure, and vocal tract shaping^(9,10)^. Glottal closure strength varies across musical styles, reflected in the aerodynamic measures of voice production, especially in Psub and transglottic airflow^(7,11,12)^.

Lung capacity directly contributes to Psub control, being crucial for precise vocal emission at varying f_0_, as it allows efficient control of expiratory airflow and Psub during singing^(13)^. The literature indicates that singers have greater vital lung capacity than non-singers, which may favor sustained vocal emission with greater physiological efficiency^(13)^. It is very important to evaluate the aerodynamic measures of singers, as variations between registers and, consequently, at different f_0_ directly influence airflow, Psub, glottal resistance, and phonatory efficiency, central elements in healthy voice production and artistic performance of the singing voice^(14)^.

This research aimed to analyze aerodynamic measures at usual, low, and high frequencies in male and female singers without voice changes.

This study is justified by the need to understand the aerodynamic mechanisms in singers based on emissions at usual, low, and high frequencies to identify specific vocal needs in singing and support the development of individualized and effective strategies for improving and training the singing voice.

## METHODS

This is a cross-sectional, analytical, observational study, approved by the Research Ethics Committee of the Federal University of Minas Gerais (UFMG) under approval number 1.229.521. All individuals were informed about the study objectives and procedures and signed an informed consent form after agreeing to them.

The inclusion criteria for the research were singers aged 18 to 55 years, without vocal complaints or changes, defined by speech-language-hearing evaluation, and with a normal larynx, as determined by otolaryngological evaluation. Participants who smoked, were pregnant, or were menstruating were excluded.

All participants underwent speech-language-hearing and otolaryngological evaluation for sample selection. The former consisted of an auditory-perceptual evaluation (APE) of voice, based on consensus between two speech-language-hearing pathologists specializing in voice with more than 5 years of experience in the field. The APE evaluated the usual sustained /a/ vowel emission, considering the auditory-perceptual parameter of the Grade of hoarseness of the voice (G). Participants without vocal deviation (G_0_) were eligible.

Participants answered the following questions to determine the absence of vocal complaints:

1. Do you have any difficulty or discomfort with your singing voice?
2. Do you have any difficulty or discomfort with your speaking voice?
3. Do you think your voice has suffered changes?

The study included singers who answered negatively to all three questions.

An otolaryngologist assessed larynges with a 70° rigid laryngoscope coupled to a 300 W Xenon light source (KayPentax^®^, Lincoln Park, New Jersey) and a high-speed color videolaryngoscopy system, model 9710.

Image resolution was set to 512 x 512 pixels with an 8-bit RGB color mode. Normal larynges were considered those whose examinations found no VF lesions, with complete glottal closure or, in the case of women, with a posterior triangular gap, considered physiological^(15)^.

The group was selected from a singing school and divided according to their self-reported musical genre and experience as a singer. Hence, 70% of women stated that their musical genre was popular singing, with experience ranging from 4 to 12 years (mean of 7.3 years; SD = 2.38); the others reported being classical singers (30%) with 3 to 10 years of experience (mean of 6.6 years; SD = 2.40).

As for men, 63% reported being popular singers with an experience of 5 to 13 years (mean of 7.5 years; SD = 2.38); the others reported being opera singers with experience ranging from 4 to 15 years (mean of 6.9 years; SD = 2.94).

The study selected 60 individuals: 30 males, aged 19 to 48 years (mean: 28.6 years; SD = 7.16), and 30 females, aged 18 to 36 years (mean: 26.1 years; SD = 5.59).

### Aerodynamic evaluation

Aerodynamic measures and acoustic measures of f_0_ of speech were evaluated with the Kay Pentax^®^ CSL software, model 6103, Lincoln Park, NJ, USA, Phonatory Aerodynamic System (PAS) module, installed on a Dell Optiplex GX260 computer with a professional sound card, Direct Sound brand, in the Observatory of Speech-Language-Hearing Functional Health at UFMG’s Medical School (OSF/UFMG).

For these measurements, participants were instructed to emit the syllable /pá/ at their usual frequency and intensity, seven consecutive times in a single exhalation. Aerodynamic measures were recorded with a silicone facial mask placed over the participant's mouth. The mask was coupled to a device connected to a pressure transducer, and intraoral pressure was measured with a small-diameter polyethylene catheter inserted into the mask through a lateral orifice and positioned in the central part of the participant's tongue. The other end of the catheter was connected to a pressure transducer.

The acoustic measure of f_0_ was captured with a microphone attached to the back of the mask. All the transducer and microphone signals were sent to the CSL program for analysis.

The following parameters were selected for aerodynamic measure analysis, with their respective reference values ​​for women and men, as reported in the Kay Pentax^®^ CSL Program manual^(16)^.

- Peak air pressure: this is the highest air pressure value in one or more plosive syllables, measured in cmH_2_O (6.65 cmH_2_O for women and 7.55 cmH_2_O for men).
- Mean peak air pressure: measured in cmH_2_O (5.57 cmH_2_O for women and 6.058 cmH_2_O for men).
- Mean airflow during vocalization: the quotient between the total volume of exhaled air and the duration of the voiced segments, measured in liters/second (0.11 l/s for women and 0.12 l/s for men).
- Aerodynamic power: the product of the average peak air pressure value, the voiced airflow, and 0.09806, measured in watts (0.06 W for women and 0.09 W for men).
- Aerodynamic resistance: the result of the average air pressure divided by the voiced airflow, measured in cmH_2_O/liters/second (55.18 cmH_2_O/l/s for women and 52.60 cmH_2_O/l/s for men).
- Acoustic impedance: the result of the average air pressure divided by the voiced airflow, measured in dyne seconds/cm^5^ (56.27 dyn s/cm^5^ for women and 53.64 dyn s/cm^5^ for men).
- Aerodynamic efficiency: dimensionless value, defined in parts per million (ppm). It is the result of dividing the acoustic power by the aerodynamic power (103.66 ppm for women and 45.81 ppm for men).

### Determining the fundamental frequency (f_0_)

Participants were instructed to perform a 5-minute vocal warm-up immediately before starting vocal emission collection. They could warm up as they wished, including exercises they normally used in singing vocal practice (e.g., musical scale sequences, lip or tongue vibration, semi-occluded vocal tract exercises). This procedure was used to ensure vocal comfort and preserve the singers' usual preparation routine. Immediately afterwards, they were asked to sustain the vowel /a/ at their usual, high, and low frequencies.

All participants were instructed to emit their usual frequency by sustaining the vowel /a/ at speech frequency, as comfortably as possible. The mean usual frequency was 197.56 Hz (152.78 to 251.98 Hz; SD = 19.73) in the women's group and 166.75 Hz (96.94 to 221.07 Hz; SD = 31.87) in the men's group.

Participants were also instructed to emit high frequency by producing the vowel /a/ one octave higher than usual. They were given an auditory stimulus with the musical note corresponding to the frequency identified in the high-frequency emission, using a CxL Key Flexible Roll Up Piano Synthesizer musical keyboard as a tone reference. The mean high frequency was 449.64 Hz (287.85 to 590 Hz; SD = 63.95) for women and 180.71 Hz (121.87 to 246.45 Hz; SD = 40.46) for men.

As for low frequency, they were instructed to emit the vowel /a/ up to three tones below the usual, also with the aid of the auditory stimulus, using the keyboard as a tone reference. The mean low frequency was 186.47 Hz (126.43 to 245.71 Hz; SD = 32.38) in the women's group and 147.28 Hz (80.95 to 219.77 Hz; SD = 39.38) in the men's group.

**Figure 1:**
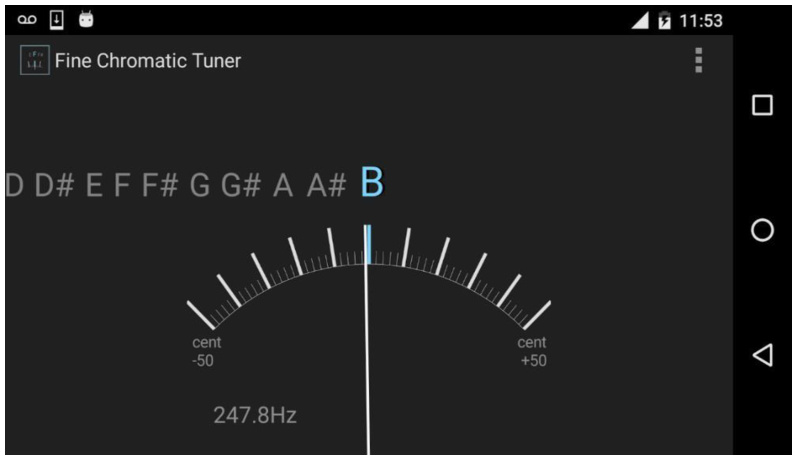
Fine Chromatic Tuner application for mobile phones

The researchers monitored the frequency of the participants' vocal emissions using the Fine Chromatic Tuner application (Figure 1).

### Data analysis

Statistical data analysis was performed using the Minitab^®^19 statistical software. First, a descriptive analysis of the data was conducted using measures of central tendency and dispersion. Then, the responses of the aerodynamic measures at different frequencies were compared with the parametric ANOVA test for repeated measures or the non-parametric Friedman test for variables with asymmetrical distribution. Analyses considered the 95% confidence level.

## RESULTS

Women had higher aerodynamic efficiency values ​​in usual emissions and higher high-pitched emission values ​​than low-pitched ones (p < 0.001) (Table 1).

**Table 1:** Comparisons of aerodynamic measures of female singers in usual, high, and low-frequency emissions

MEASURES		MINIMUM	MAXIMUM	MEAN	SD	P-Value
Peak air pressure (cmH_2_O)	*Usual*	4.82	13.94	9.46	2.88	0.334 ^Ɨ^
	*High*	4.82	13.9	9.45	2.47	
	*Low*	4.51	13.94	8.60	2.32	
Mean peak air pressure (cmH_2_O)	*Usual*	4.41	13.49	7.86	2.75	1.000 ^Ɨ Ɨ^
	*High*	4.41	13.49	7.91	2.44	
	*Low*	4.11	13.49	7.89	2.19	
Mean airflow during vocalization (l/s)	*Usual*	0.08	0.33	0.19	0.07	0.865 ^Ɨ Ɨ^
	*High*	0.08	0.33	0.19	0.06	
	*Low*	0.08	0.32	0.18	0.06	
Aerodynamic power (W)	*Usual*	0.038	0.44	0.16	0.11	0.775 ^Ɨ Ɨ^
	*High*	0.038	0.44	0.17	0.091	
	*Low*	0.055	0.441	0.15	0.07	
Aerodynamic resistance (cmH_2_O/(l/s)	*Usual*	19.95	67.47	41.63	12.13	0.739 ^Ɨ^
	*High*	19.95	89.22	43.39	15.94	
	*Low*	17.95	67.57	40.51	14.993	
Acoustic impedance (dyn s/cm^5^)	*Usual*	20.35	68.80	42.53	12.43	0.975 ^Ɨ^
	*High*	20.35	70.98	43.31	14.33	
	*Low*	18.3	68.9	42.73	15.809	
Aerodynamic efficiency (ppm)	*Usual*	90.39	376.30	211.94	76.78	**<0.001** ^Ɨ^
	*High*	80.54	343.19	168.34	66.24	
	*Low*	55.66	201.41	122.04	46.86	

Ɨ ANOVA test;

Ɨ Ɨ Friedman test; bold: p < 0.05]

**Caption:** cmH_2_O: centimeters of water; SD: standard deviation; dyn s/cm: dyne second/centimeter; l/s: liters per second; ppm: parts per million

The variation of f_0_ in the voice causes greater changes in the aerodynamic parameters of males. In high-pitched emissions, men had higher peak Psub values ​​(p = 0.035); these emissions resulted in greater aerodynamic resistance (p = 0.001). The high-pitched frequency also affected acoustic impedance parameters (p = 0.001) and aerodynamic efficiency (p < 0.001), which presented significantly higher values ​​when compared to low-pitched emissions (Table 2).

**Table 2:** Comparisons of aerodynamic measures of male singers in usual, high, and low-frequency emissions

MEASURES		MINIMUM	MAXIMUM	MEAN	SD	P-Value
Peak air pressure (cmH_2_O)	*Usual*	4.41	13.94	8.56	3.06	**0.035** ^Ɨ Ɨ^
	*High*	5.4	21.02	9.19	3.62	
	*Low*	4.41	13.94	8.31	2.67	
Mean peak air pressure (cmH_2_O)	*Usual*	3.81	13.49	8.94	2.82	0.139 ^Ɨ^
	*High*	5.15	19.99	9.54	3.39	
	*Low*	3.81	13.49	8.03	2.52	
Mean airflow during vocalization (l/s)	*Usual*	0.08	0.42	0.17	0.075	0.241 ^Ɨ^
	*High*	0.05	0.35	0.16	0.074	
	*Low*	0.08	0.32	0.19	0.058	
Aerodynamic power (W)	*Usual*	0.038	0.44	0.15	0.09	0.498 ^Ɨ Ɨ^
	*High*	0.031	0.533	0.15	0.118	
	*Low*	0.038	0.44	0.15	0.07	
Aerodynamic resistance (cmH_2_O/l/s)	*Usual*	12.76	67.75	45.25	13.51	**0.001** ^Ɨ^
	*High*	18.67	113.25	58.25	26.755	
	*Low*	12.76	89.22	39.35	16.039	
Acoustic impedance (dyn s/cm^5^)	*Usual*	13.02	69.12	42.53	14.303	**0.001** ^Ɨ^
	*High*	19.04	134.89	57.00	31.609	
	*Low*	13.02	128.92	41.75	22.834	
Aerodynamic efficiency (ppm)	*Usual*	101.09	324.12	167.00	76.26	**<0.001** ^Ɨ Ɨ^
	*High*	131.32	1206.55	470.40	311.82	
	*Low*	42.31	800.32	167.03	164.16	

Ɨ ANOVA test;

Ɨ Ɨ Friedman test; bold: p < 0.05

**Caption:** cmH_2_O: centimeters of water; SD: standard deviation; dyn s/cm: dyne second/centimeter; l/s: liters per second; ppm: parts per million

## DISCUSSION

Breathing is the basis of voice production in both speaking and singing voices^(17)^. Different breathing behaviors can significantly impact the vocal result, due to the systemic interdependence between phonation and breathing^(6,18)^. This is because aerodynamic energy is converted into acoustic energy through glottal closure combined with their resistance to the exhaled airflow and the air pressure in the lungs after inspiration and airflow, which directly influence the VF vibration conditions^(7,8)^.

In the singing voice, the choices of respiratory strategies and muscle contraction adjustments vary according to the singer's musical style and aesthetic preferences^(11)^. A study with 60 professional singers found that sopranos have lower lung volume and vital capacity and higher f_0_ emission, while basses have higher volumes in the emission of sounds in low ranges, requiring more controlled and sustained airflow, which correlates with a higher total lung volume^(12)^.

This study found that women have greater aerodynamic efficiency in usual emissions – i.e., they can convert a greater amount of acoustic energy from a smaller amount of aerodynamic energy in usual emissions, followed by high-pitched emissions. A study^(19)^ showed that male and female singers have lower airflow and lower Psub than non-singers, indicating greater aerodynamic efficiency, which reflects refined respiratory control and effective use of lung energy to convert aerodynamic power into acoustic sound^(19)^.

The aeromechanical mechanisms of VF vibratory behavior are substantially different between registers^(20)^. In a study with 20 participants (10 men and 10 women), the airflow rate was three times higher and the Psub 1.5 times higher in the modal register than in the baseline register^(20)^.

It can be assumed that women produce usual sounds more efficiently and low sounds less efficiently than men, with a lower aerodynamic requirement in f_0_ variation. A reduction in pressure and flow is observed in the low register, consistent with less efficient phonation. Psub and flow tend to increase in high f_0_, requiring more respiratory support and precise control of glottal closure^(21)^. A study with nine sopranos demonstrated high Psub and VF closure at high frequencies^(21)^. Women have lower VF vibratory mass, with a consequent lower demand for flow and pressure for oscillation, which favors higher-pitched emissions^(22)^.

Men, on the other hand, have greater aerodynamic changes when performing variations in voice f_0_ towards the high register, with an increase in Psub, aerodynamic resistance, and acoustic impedance. It is worth highlighting that the increase in Psub may be the aspect that influenced the aerodynamic resistance and acoustic impedance measures, since these two aerodynamic parameters are dependent on Psub, which is the driving force for phonation^(23)^.

A study with 20 singers and 20 non-singers reported that Psub is considered determinant for increasing vocal intensity, while subglottal airflow remains relatively constant^(19)^. A study with male singers (baritones/basses) demonstrated that the increase in Psub is strongly related to greater phonatory efficiency^(24)^.

It was also observed that, although high-frequency emission in men demands greater aerodynamic efforts, the increase in Psub occurs with a concomitant increase in aerodynamic efficiency values. This suggests that men efficiently convert aerodynamic energy into acoustic energy when producing high-pitched sounds. A study^(24)^ showed that male singers have phonatory efficiency, with significantly higher Psub when singing at high frequencies, with optimized vocal technique^(24)^.

This study showed that f_0_ variation on an ascending scale generates a much greater aerodynamic demand in men than in women, and that men, as already described in the literature^(17)^, must increase Psub considerably in comparison with women to be able to perform high notes, which also increases other measures related to Psub, such as resistance and impedance^(17)^.

F_0_ is an essential parameter in the evaluation of the singing voice, especially due to its direct influence on acoustic and aerodynamic parameters of voice production, with wide variation in singing depending on the vocal register and technique used^(9)^. Differences in aerodynamic parameters between men and women may be related to the anatomical characteristics of the larynx between the sexes^(22)^. Singers learn to control vocal structures with greater capacity and effectiveness using the laryngeal intrinsic and extrinsic muscles, the hyoid-larynx complex, VF vibration patterns, and the dimensions of the vocal tract^(25,26)^.

It is already known that correct muscle recruitment combined with an appropriate breathing pattern provides greater coordination of aerodynamic measures during singing^(27)^. Airflow imbalance or any other underlying pulmonary problem can severely affect the proper phonatory control and sound quality of singers^(28)^. Thus, knowledge about the functioning of the respiratory system and the influence of aerodynamic measures on phonation is extremely important. The literature^(29,30)^ has been concerned with establishing instrumental aeroacoustic measures as part of a multidimensional diagnostic approach for monitoring functional changes in the singing voice and developing preventive and rehabilitative care in singing^(29,30)^.

Although the research has been limited to the aerodynamic process of singers, understanding that the voice results from the interaction between breathing, source and filter is crucial for the student's learning process, in the case of singing teachers, and the patients’ habilitation/rehabilitation, in the case of speech-language-hearing pathologists. As limitations of this research, we highlight that musical styles among singers were not controlled in the sample. Future research is needed to analyze the relationship between musical styles and respiratory aspects in the emission of different frequencies in men and women, aiming not only to establish an ideal pattern that helps them use their vocal potential to the fullest during their performances, but also to promote longevity and health of the entire vocal apparatus.

## CONCLUSION

The variation in f_0_ of the voice causes greater modifications in the aerodynamic parameters of male singers. Female singers had higher aerodynamic efficiency values in usual voice production. Male singers had higher values ​​of peak air pressure, aerodynamic resistance, acoustic impedance, and aerodynamic efficiency in high-pitched voice production.
