# Supplementary material for: Aerodynamic measurements in singers at usual, low, and high frequencies
Source: Codas. 2026 May 11;38(3):e20250325. doi: 10.1590/2317-1782/e20250325en (PMC13193190; doi:10.1590/2317-1782/e20250325en)
Supplement: Translation

# Translation: Medidas aerodinâmicas em cantores nas frequências habituais, graves e agudas

**DOI:** 10.1590/2317-1782/e20250325pt

**Authors:** Dâmaris Poliana Lacerda Vieira, Viviane Souza Bicalho Bacelete, Bárbara Pereira Lopes, Patrícia de Freitas Lopes Genilhú, Ana Cristina Côrtes Gama

**Affiliations:** 1 Programa de Pós-graduação em Ciências Fonoaudiológicas, Departamento de Fonoaudiologia, Faculdade de Medicina, Universidade Federal de Minas Gerais – UFMG - Belo Horizonte (MG), Brasil.

**Keywords:** Acústica, Música, Canto, Respiração, Voz

## Abstract

**Objetivo:**

Analisar as medidas aerodinâmicas nas frequências habituais, graves e agudas em homens e mulheres cantores sem alterações vocais.

**Método:**

Trata-se de uma pesquisa do tipo observacional analítica transversal. Participaram da pesquisa 30 homens e 30 mulheres cantores, com idade entre 19 e 48 anos. Foi realizada a medida dos parâmetros aerodinâmicos de pressão aérea, fluxo de ar expirado e vozeado, volume expiratório, potência e resistência aerodinâmica, impedância acústica e eficiência aerodinâmica por meio da emissão da sílaba /pá/ por sete vezes consecutivas em frequências habituais, graves e agudas em intensidade habitual.

**Resultados:**

As modificações nas medidas aerodinâmicas durante a emissão de diferentes frequências da voz foram mais evidentes em cantores do sexo masculino. Cantores homens apresentaram, nas emissões agudas, maiores valores de pico de pressão aérea, resistência aerodinâmica, impedância acústica e eficiência aerodinâmica enquanto que as mulheres cantoras apresentaram maiores valores de eficiência aerodinâmica na emissão habitual, seguida das emissões agudas.

**Conclusão:**

A variação de frequência fundamental na voz provoca maiores modificações nos parâmetros aerodinâmicos nos homens cantores. As mulheres cantoras apresentaram maiores valores de eficiência aerodinâmica na emissão habitual.

## INTRODUÇÃO

Diversas teorias sobre a produção da voz surgiram no decorrer do tempo, destacando-se a Teoria Mioelástica Aerodinâmica, de Van Den Berg, que inter-relaciona a elasticidade da musculatura laríngea com a força aerodinâmica da respiração, formando um sistema interdependente^(1)^. Ignorar qualquer uma das partes deste sistema pode comprometer o equilíbrio na produção vocal e aumentar o risco de desenvolvimento de distúrbios vocais, por quebra da homeostase^(1,2)^.

A utilização da avaliação aerodinâmica na clínica vocal tem se tornado cada vez mais comum, uma vez que essas medidas fazem parte do protocolo de recomendação de análise instrumental da voz sugerido pela ASHA (*American Speech-Language-Hearing Association*) e fornecem valiosas informações funcionais sobre a inter-relação entre os sistemas fonatório e respiratório^(2,3)^. Volumes e capacidades pulmonares, pressão aérea subglótica (Psub), fluxo de ar laríngeo, resistência laríngea e relação s/z são medidas disponíveis para uso na clínica vocal e úteis por fornecerem detalhes da fisiologia da fonação^(2-4)^.

O modo interdependente de funcionamento do sistema fonatório permite compreender como a atividade dos músculos adutores das pregas vocais (PPVV) pode ser diretamente afetada pelo processo aerodinâmico da fonação^(1,4,5)^. A Psub resultante da compressão pulmonar por meio da musculatura expiratória e da força de fechamento das PPVV, que confere maior ou menor resistência a esse fluxo de ar transglótico, serve como fonte de energia para a vibração das PPVV e exerce influência sobre a intensidade e variações da frequência fundamental (f_0_) da voz^(6-8)^.

O canto elicia vários parâmetros físico-acústicos que são de muitas maneiras diferentes da fala^(9,10)^. Fisiologicamente, esses mecanismos se manifestam no controle e na interação entre a Psub, adução glótica e modelagem do trato vocal^(9,10)^. Entre os diferentes estilos musicais, observam-se variações na força de adução glótica, as quais se refletem nas medidas aerodinâmicas da produção vocal, especialmente na Psub e no fluxo aéreo transglótico^(7,11,12)^.

A capacidade pulmonar contribui diretamente para o controle da Psub, sendo crucial para a emissão vocal precisa em f_0_ variadas, por permitir o controle eficiente do fluxo aéreo expiratório e da Psub durante o canto^(13)^. A literatura aponta que cantores apresentam maior capacidade vital pulmonar em comparação a não cantores, o que pode favorecer a emissão vocal sustentada e com maior eficiência fisiológica^(13)^. É muito importante realizar a avaliação das medidas aerodinâmicas de cantores, pois variações entre registros e, consequentemente, em diferentes f_0_ influenciam diretamente o fluxo aéreo, Psub, resistência glótica e eficiência fonatória, elementos centrais na produção vocal saudável e no desempenho artístico da voz cantada^(14)^.

O objetivo desta pesquisa foi analisar as medidas aerodinâmicas nas frequências habituais, graves e agudas em homens e mulheres cantores sem alterações vocais.

Este estudo se justifica pela necessidade de compreender os mecanismos aerodinâmicos em cantores a partir de emissões nas frequências habituais, graves e agudas, de modo a identificar demandas específicas da voz no canto e subsidiar o desenvolvimento de estratégias individualizadas e eficazes para o aprimoramento e treinamento da voz cantada.

## MÉTODO

Trata-se de uma pesquisa observacional analítica transversal aprovada pelo Comitê de Ética em Pesquisa da Universidade Federal de Minas Gerais sob o parecer número 1.229.521. Todos os indivíduos foram informados quanto aos objetivos e procedimentos do estudo e, após concordância, assinaram o Termo de Consentimento Livre e Esclarecido (TCLE).

Os critérios de inclusão da pesquisa foram: ser cantor, entre 18 e 55 anos, sem queixas e alterações vocais definidas por avaliação fonoaudiológica e com presença de laringe normal a partir de avaliação otorrinolaringológica. Foram excluídos participantes fumantes, mulheres grávidas ou em período menstrual.

Para seleção da amostra, todos os participantes realizaram avaliação fonoaudiológica e otorrinolaringológica. A avaliação fonoaudiológica constou de avaliação perceptivo-auditiva (APA) da voz, realizada por consenso de duas fonoaudiólogas especialistas em voz com mais de cinco anos de experiência na área. A APA foi realizada a partir da avaliação da emissão da vogal /a/ sustentada de forma habitual, considerando o parâmetro perceptivo-auditivo de grau geral (G) de alteração vocal. Foram elegíveis os participantes com qualidade vocal sem desvio (G_0_).

Para determinação da ausência de queixa vocal, os participantes responderam às perguntas a seguir,

1. Você possui alguma dificuldade ou desconforto na sua voz cantada?
2. Você possui alguma dificuldade ou desconforto na sua voz falada?
3. Você acha que sua voz é alterada?

Foram incluídos na pesquisa os cantores que responderam de forma negativa às três perguntas.

A avaliação laríngea foi realizada por um médico otorrinolaringologista, utilizando laringoscópio rígido de 70°, acoplado a uma fonte de luz Xenon de 300 W (KayPentax®, Lincoln Park, New Jersey) com sistema de videolaringoscopia de alta velocidade colorido, modelo 9710.

A resolução da imagem utilizada foi 512 x 512 pixels com modo de cor de 8 bit RGB. Foram consideradas laringes normais aquelas cujos exames não apresentaram lesões em PPVV e que tinham fechamento glótico completo ou, no caso das mulheres, que apresentaram fenda triangular posterior, considerada fisiológica^(15)^.

O grupo foi selecionado em uma escola de canto e dividido de acordo com sua autorreferência sobre sua experiência como cantor e gênero musical. Das mulheres, 70% declararam ter o canto popular como seu gênero musical, com experiência variando de 4 a 12 anos (média de 7,3 anos; DP=2,38) e as demais informaram ser cantoras eruditas (30%) e possuir experiência entre 3 e 10 anos no canto erudito (média de 6,6 anos; DP=2,40).

Dos homens, 63% declararam-se cantores populares com um tempo de experiência de cinco a 13 anos (média de 7,5 anos; DP=2,38) e os demais, informaram ser cantores líricos com experiência variando entre quatro e 15 anos (média de 6,9 anos; DP=2,94).

Foram selecionados 60 indivíduos, sendo 30 do sexo masculino, com idades entre 19 e 48 anos (média 28,6 anos; DP=7,16), e 30 do sexo feminino, com idade entre 18 e 36 anos (média 26,1 anos; DP=5,59).

### Avaliação aerodinâmica

A avaliação das medidas aerodinâmicas ^e da medida acústica de f^_^0^_^da fala foram^ realizadas com uso do software CSL da Kay Pentax®, model 6103, Lincoln Park, NJ, USA, módulo Phonatory Aerodynamic System (PAS), instalado no computador da marca Dell, modelo Optiplex GX260, com placa de som profissional, marca Direct Sound do Observatório de Saúde Funcional em Fonoaudiologia da Faculdade de Medicina da UFMG (OSF/UFMG).

Para estas medidas, os participantes foram orientados a emitir a sílaba /pá/ em frequência e intensidade habituais, sete vezes consecutivas em uma única expiração. O registro das medidas aerodinâmicas realizou-se com o auxílio de uma máscara facial de silicone colocada sobre a boca do participante. A máscara foi acoplada a um dispositivo conectado a um transdutor de pressão, e a pressão intraoral foi medida por meio de um cateter de polietileno de pequeno diâmetro inserido na máscara através de um orifício lateral e posicionado na parte central da língua do participante. A outra extremidade do cateter foi conectada a um transdutor de pressão.

^A medida acústica de f^_^0^_^foi captada por meio de um microfone, acoplado^ na parte posterior da máscara. Todos os sinais dos transdutores e do microfone foram enviados ao programa CSL para análise.

Para análise das medidas aerodinâmicas, foram selecionados os seguintes parâmetros, com seus respectivos valores de referência para mulheres e homens, informados no manual do Programa CSL da Kay Pentax®^(16)^.

- Pico de pressão aérea: esta medida é o maior valor de pressão aérea observado em uma ou mais sílabas plosivas, medido em cmH_2_O (6,65 cmH_2_O para mulheres e 7,55 cmH_2_O para homens).
- Valor médio do pico de pressão aérea: medido em cmH_2_O (5,57 cmH_2_O para mulheres e 6,058 cmH_2_O para homens).
- Média do fluxo aéreo durante vocalização: quociente entre o volume total de ar expirado e a duração dos segmentos vozeados, medido em litros/segundo (0,11 l/s para mulheres e 0,12 l/s para homens).
- Potência aerodinâmica: produto entre o valor médio do pico de pressão aérea, o fluxo de ar vozeado e o valor de 0,09806, medido em watts (0,06 watts para mulheres e 0,09 watts para homens).
- Resistência aerodinâmica: definida como o resultado da média da pressão de ar dividido pelo fluxo de ar vozeado, medido em cmH_2_O/litros/segundo (55,18 cmH_2_O/l/s para mulheres e 52,60 cmH_2_O/l/s para homens).
- Impedância acústica: definida como o resultado da média da pressão de ar dividido pelo fluxo de ar vozeado, medido em dyne segundo/cm^5^ (56,27 dyn s/cm^5^para mulheres e 53,64 dyn s/cm^5^para homens).
- Eficiência aerodinâmica: valor adimensional, definido em partes por milhão (ppm). Representa o resultado da divisão entre a potência acústica e a potência aerodinâmica (103,66 ppm para mulheres e 45,81 ppm para homens).

### Determinação da frequência fundamental (f_0_)

Os participantes foram orientados a realizar um aquecimento vocal de cinco minutos imediatamente antes do início da coleta das emissões vocais. O aquecimento foi de livre escolha, contemplando exercícios usuais da prática vocal cantada de cada participante (p. ex., sequências de escalas musicais, vibração de lábios ou língua, exercícios de trato vocal semiocluído). Essa opção foi adotada para garantir conforto vocal e preservar a rotina habitual de preparo dos cantores. Logo em seguida, foram solicitados a emitir de forma sustentada a vogal /a/ nas frequências habitual, aguda e grave.

Para a emissão da frequência habitual, todos os participantes foram orientados a sustentar a vogal /a/ na frequência da fala, da forma mais confortável possível. No grupo de mulheres, o valor médio de frequência habitual foi de 197,56 Hz (152,78 a 251,98 Hz; DP=19,73), e no grupo de homens foi de 166,75 Hz (96,94 a 221,07 Hz; DP=31,87).

A emissão da frequência aguda foi realizada a partir da orientação para que os participantes produzissem a vogal /a/ uma oitava acima da habitual. Neste momento, foi dado ao participante um estímulo auditivo, referente à nota musical correspondente à frequência identificada na emissão aguda, utilizando-se um teclado musical da marca CxL Key Flexível Roll Up Piano Sintetizador para referência de tonalidade. O valor da média da frequência aguda das mulheres foi de 449,64 Hz (287,85 a 590 Hz; DP=63,95) e dos homens, 180,71 Hz (121,87 a 246,45 Hz; DP=40,46).

Na emissão da frequência grave, os indivíduos foram orientados a emitir a vogal /a/ em frequência de até três tons abaixo da habitual, também com o auxílio do estímulo auditivo, utilizando-se o teclado para referência de tonalidade. A frequência grave teve valor médio no grupo de mulheres de 186,47 Hz (126,43 a 245,71 Hz; DP=32,38) e, no grupo de homens, 147,28 Hz (80,95 a 219,77 Hz; DP=39,38).

^As pesquisadoras monitoraram a frequênciadas emissões vocais dos^ participantes, utilizando o aplicativo *Fine Chromatic Tuner* (Figura 1).

**Figura 1:** Aplicativo *Fine Chromatic Tuner* para celulares

### Análise dos dados

A análise estatística dos dados foi realizada por meio do programa estatístico Minitab®19. Primeiramente, foi realizada uma análise descritiva dos dados com medidas de tendência central e dispersão. Posteriormente, para comparação entre as respostas das medidas aerodinâmicas em diferentes frequências, foi utilizado o teste paramétrico ANOVA para medidas repetidas ou o teste não-paramétrico de Friedman nas variáveis com distribuição assimétrica. Considerou-se o nível de confiança de 95%.

## RESULTADOS

As mulheres apresentaram maiores valores de eficiência aerodinâmica na emissão habitual e valores de emissões agudas superiores aos das graves (p<0,001) (Tabela 1).

**Tabela 1:** Comparações das medidas aerodinâmicas de mulheres cantoras em emissões de frequência habitual, aguda e grave

MEDIDAS		MÍNIMO	MÁXIMO	MÉDIA	DP	P-Valor
Pico de pressão aérea (cm H_2_O)	*Habitual*	4,82	13,94	9,46	2,88	0,334^Ɨ^
	*Agudo*	4,82	13,9	9,45	2,47	
	*Grave*	4,51	13,94	8,60	2,32	
Valor médio do pico de pressão aérea (cm H_2_O)	*Habitual*	4,41	13,49	7,86	2,75	1,000 ^Ɨ Ɨ^
	*Agudo*	4,41	13,49	7,91	2,44	
	*Grave*	4,11	13,49	7,89	2,19	
Média do fluxo aéreo durante a vocalização (l/s)	*Habitual*	0,08	0,33	0,19	0,07	0,865 ^Ɨ Ɨ^
	*Agudo*	0,08	0,33	0,19	0,06	
	*Grave*	0,08	0,32	0,18	0,06	
Potência aerodinâmica (watts)	*Habitual*	0,038	0,44	0,16	0,11	0,775 ^Ɨ Ɨ^
	*Agudo*	0,038	0,44	0,17	0,091	
	*Grave*	0,055	0,441	0,15	0,07	
Resistência aerodinâmica (cm H_2_O/ (l/s)	*Habitual*	19,95	67,47	41,63	12,13	0,739 ^Ɨ^
	*Agudo*	19,95	89,22	43,39	15,94	
	*Grave*	17,95	67,57	40,51	14,993	
Impedância acústica (ds/cm^5^)	*Habitual*	20,35	68,80	42,53	12,43	0,975 ^Ɨ^
	*Agudo*	20,35	70,98	43,31	14,33	
	*Grave*	18,3	68,9	42,73	15,809	
Eficiência aerodinâmica (ppm)	*Habitual*	90,39	376,30	211,94	76,78	**<0,001** ^Ɨ^
	*Agudo*	80,54	343,19	168,34	66,24	
	*Grave*	55,66	201,41	122,04	46,86	

Ɨ teste ANOVA;

Ɨ Ɨ *teste de Friedman*; negrito: p < que 0,05

**Legenda:** cm H_2_O:centímetros de água; DP: desvio padrão; dyne/centímetro; l/s: litros por segundo; ppm: parte por milhão

A variação de f_0_ na voz provoca maiores modificações nos parâmetros aerodinâmicos dos indivíduos do sexo masculino. Observou-se que, nas emissões agudas, os homens apresentaram maiores valores de pico de Psub (p=0,035), e tais emissões resultaram em maior resistência aerodinâmica (p=0,001). A frequência aguda também impactou os parâmetros de impedância acústica (p=0,001) e eficiência aerodinâmica (p<0,001), que apresentaram valores significativamente superiores quando comparados às emissões graves (Tabela 2).

**Tabela 2:** Comparações das medidas aerodinâmicas de homens cantores em emissões de frequência habitual, aguda e grave

MEDIDAS		MÍNIMO	MÁXIMO	MÉDIA	DP	P-Valor
Pico de pressão aérea (cm H_2_O)	*Habitual*	4,41	13,94	8,56	3,06	**0,035** ^Ɨ Ɨ^
	*Agudo*	5,4	21,02	9,19	3,62	
	*Grave*	4,41	13,94	8,31	2,67	
Valor médio do pico de pressão aérea (cm H_2_O)	*Habitual*	3,81	13,49	8,94	2,82	0,139 ^Ɨ^
	*Agudo*	5,15	19,99	9,54	3,39	
	*Grave*	3,81	13,49	8,03	2,52	
Média do fluxo aéreo durante a vocalização (l/s)	*Habitual*	0,08	0,42	0,17	0,075	0,241 ^Ɨ^
	*Agudo*	0,05	0,35	0,16	0,074	
	*Grave*	0,08	0,32	0,19	0,058	
Potência aerodinâmica (watts)	*Habitual*	0,038	0,44	0,15	0,09	0,498 ^Ɨ Ɨ^
	*Agudo*	0,031	0,533	0,15	0,118	
	*Grave*	0,038	0,44	0,15	0,07	
Resistência aerodinâmica (cm H_2_O /l/s)	*Habitual*	12,76	67,75	45,25	13,51	**0,001** ^Ɨ^
	*Agudo*	18,67	113,25	58,25	26,755	
	*Grave*	12,76	89,22	39,35	16,039	
Impedância acústica (ds/cm^5^)	*Habitual*	13,02	69,12	42,53	14,303	**0,001** ^Ɨ^
	*Agudo*	19,04	134,89	57,00	31,609	
	*Grave*	13,02	128,92	41,75	22,834	
Eficiência aerodinâmica (ppm)	*Habitual*	101,09	324,12	167,00	76,26	**<0,001** ^Ɨ Ɨ^
	*Agudo*	131,32	1206,55	470,40	311,82	
	*Grave*	42,31	800,32	167,03	164,16	

Ɨ teste ANOVA;

Ɨ Ɨ *teste de Friedman*; negrito: p < que 0,05

**Legenda:** cm H_2_O: centímetros de água; DP – desvio padrão; ds/cm: dyne/centímetro; l/s: litros por segundo; ppm: parte por milhão

## DISCUSSÃO

A respiração é a base da produção vocal, tanto na voz falada como na voz cantada^(17)^. Diferentes comportamentos respiratórios podem impactar expressivamente o resultado vocal, devido à interdependência sistêmica entre fonação e respiração^(6,18)^. Isso porque a conversão da energia aerodinâmica em acústica ocorre por meio da adução glótica somada à resistência que estas oferecem ao fluxo de ar expirado e a pressão aérea existente nos pulmões após a inspiração e o fluxo de ar, que influem diretamente nas condições de vibração das PPVV^(7,8)^.

Na voz cantada, as escolhas das estratégias respiratórias bem como dos ajustes de contração da musculatura envolvida irão variar de acordo com o estilo musical do cantor e de suas preferências estéticas^(11)^_. Em uma pesquisa com 60 cantores profissionais, observou-se que sopranos apresentam menores volume pulmonar e capacidade vital e emissão em maior f _0_ , enquanto que baixos apresentam maiores volumes em emissão de sons em faixas graves, que requerem fluxo aéreo mais controlado e sustentado, o que se correlaciona com maior volume pulmonar total_^(12)^_._

Neste estudo, foi possível observar que as mulheres apresentam maior eficiência aerodinâmica nas emissões habituais, ou seja, são capazes de converter uma maior quantidade de energia acústica a partir de uma menor quantidade de energia aerodinâmica nas emissões habituais, seguidas das agudas. Uma pesquisa^(19)^ evidenciou que homens e mulheres cantores apresentam menor fluxo aéreo e menor Psub do que não cantores, indicando maior eficiência aerodinâmica, que reflete um controle respiratório refinado e uso eficaz da energia pulmonar para converter potência aerodinâmica em som acústico^(19)^.

Os mecanismos aeromecânicos do comportamento vibratório das PPVV são substancialmente diferentes entre os registros^(20)^. Em uma pesquisa com 20 participantes (10 homens e 10 mulheres), no registro modal a taxa de fluxo de ar foi três vezes maior e a Psub 1,5 vezes maior em comparação ao registro basal^(20)^.

É possível supor que as mulheres são mais eficientes na produção de sons habituais e menos eficientes na produção de sons graves, apresentando uma menor exigência aerodinâmica na variação de f_0_, quando comparadas aos homens. No registro grave, observa-se redução de pressão e fluxo, compatível com uma fonação menos eficiente. Em f_0_ agudas, a Psub e o fluxo tendem a aumentar, exigindo mais suporte respiratório e controle preciso do fechamento glótico^(21)^. Um estudo com nove sopranos demonstrou Psub elevada e fechamento das PPVV em altas frequências^(21)^. As mulheres apresentam menor massa vibratória de PPVV, com consequente menor demanda de fluxo e pressão para oscilação, o que favorece emissões mais agudas^(22)^.

Já os homens apresentam maiores modificações aerodinâmicas quando realizam variações de f_0_ da voz para o agudo, com aumento da Psub, resistência aerodinâmica e impedância acústica. Vale destacar que o aumento da Psub pode ser o aspecto que influenciou as medidas de resistência aerodinâmica e impedância acústica, já que estes dois parâmetros aerodinâmicos são dependentes da Psub, que é a força motriz para fonação^(23)^.

Estudo com 20 cantores e 20 não cantores reportou que a Psub é considerada determinante para o aumento da intensidade vocal, enquanto o fluxo aéreo subglótico mantém-se relativamente constante^(19)^_._ Um estudo com cantores homens (barítonos/baixos) demonstrou que o aumento da Psub está fortemente relacionado a uma maior eficiência fonatória^(24)^.

Também foi possível observar que, apesar de a emissão em frequência aguda nos homens demandar maiores esforços aerodinâmicos, o aumento da Psub ocorre com um aumento concomitante dos valores da eficiência aerodinâmica, sugerindo que os homens, ao produzirem sons agudos, convertem de forma eficiente a energia aerodinâmica em energia acústica. Uma pesquisa^(24)^ evidenciou que homens cantores apresentam eficiência fonatória, com Psub significativamente maior ao cantar em frequências agudas, evidenciando técnica vocal otimizada^(24)^.

Esse estudo mostrou que a variação da f_0_ em uma escala ascendente gera uma demanda aerodinâmica muito maior em homens do que em mulheres, sendo que nos homens, conforme já descrito na literatura^(17)^, é necessário que a Psub aumente consideravelmente, quando comparado às mulheres, para que seja possível a execução de notas agudas, o que também levará ao aumento de outras medidas que se relacionam à Psub , como a resistência e a impedância^(17)^.

A f_0_ é um parâmetro essencial na avaliação da voz cantada, especialmente por sua influência direta nos parâmetros acústicos e aerodinâmicos da produção vocal, com variação ampla no canto dependendo do registro vocal e da técnica utilizada^(9)^. As diferenças nos parâmetros aerodinâmicos entre homens e mulheres podem estar relacionadas às características anatômicas da laringe entre os sexos^(22)^. Cantores aprendem a controlar as estruturas vocais com mais capacidade e eficácia usando os músculos intrínsecos e extrínsecos da laringe, o complexo hioide-laringe, os padrões de vibração das PPVV e dimensões do trato vocal^(25,26)^.

Já se sabe que o recrutamento correto da musculatura somado ao padrão respiratório adequado proporciona maior coordenação das medidas aerodinâmicas durante o canto^(27)^. Um desequilíbrio no fluxo de ar ou qualquer outro problema pulmonar subjacente pode afetar gravemente o controle fonatório adequado e a qualidade do som de cantores^(28)^. Por esse motivo, é de extrema importância o conhecimento sobre o funcionamento do sistema respiratório e da influência das medidas aerodinâmicas na fonação. A literatura^(29,30)^ tem se preocupado com estabelecimento de medidas instrumentais aerocústicas como parte de uma abordagem diagnóstica multidimensional para monitoramento de mudanças funcionais na voz cantada e desenvolvimento de cuidados preventivos e reabilitadores no canto^(29,30)^.

Embora a pesquisa tenha se limitado ao processo aerodinâmico de cantores e cantoras, compreender que a voz é resultado da interação entre respiração, fonte e filtro é crucial para o processo de aprendizagem do aluno, no caso dos professores de canto, ou da habilitação/reabilitação dos pacientes, no caso dos fonoaudiólogos.

Como limitações desta pesquisa, destacamos o fato de os estilos musicais entre cantores não terem sido controlados na amostra. Pesquisas futuras são necessárias para analisar a relação entre os estilos musicais e os aspectos respiratórios na emissão de diferentes frequências em homens e mulheres, visando não apenas estabelecer um padrão ideal que auxilie esses indivíduos a usarem o máximo de seu potencial vocal durante suas performances, mas também promover longevidade e saúde de todo o aparato fonador.

## CONCLUSÃO

A variação de frequência fundamental na voz provoca maiores modificações nos parâmetros aerodinâmicos dos homens cantores. Cantoras apresentaram maiores valores de eficiência aerodinâmica na emissão habitual. Os cantores apresentaram, nas emissões agudas, maiores valores de pico de pressão aérea, resistência aerodinâmica, impedância acústica e eficiência aerodinâmica.
